# Athletic identity and depressive symptom risk in college students: a machine learning approach to identify at-risk profiles and protective correlates

**DOI:** 10.3389/fpsyg.2026.1764766

**Published:** 2026-06-05

**Authors:** Deqing Chang

**Affiliations:** Zhengzhou University of Science and Technology, Zhengzhou, China

**Keywords:** athletic identity, college students, depression risk, depressive symptoms, machine learning, sports participation

## Abstract

The relationship between athletic participation and depressive symptom risk among college students remains complex and contested, with prior research yielding inconsistent findings regarding whether sports involvement serves as a protective correlate or a correlate of psychological vulnerability. Using cross-sectional survey data from 795 undergraduate students at a large public university in the northeastern United States, this study employed machine learning methods to examine how athletic identity profiles, sports participation patterns, and related psychosocial correlates were associated with depressive symptoms. We specifically aimed to identify distinct athletic identity profiles, evaluate whether these profiles improved depression-risk prediction, and examine whether risky health behaviors and masculine norm variables statistically accounted for part of the observed associations. Using unsupervised clustering algorithms, we identified four distinct athletic identity profiles characterized by varying combinations of jock identity strength, athlete identity strength, and sport goal orientation. Gradient boosting classification models were then developed to predict elevated depressive symptoms, achieving robust predictive performance validated through five-fold cross-validation. Feature importance analysis revealed that the association between athletic participation and depressive symptom risk is highly context-dependent, moderated by factors including gender conformity norms, primary sport type, competitive level, and the balance between task and ego orientation in sports. Notably, strong athlete identity characterized by intrinsic motivation and task mastery orientation was associated with lower depression risk, whereas strong jock identity characterized by status-seeking and conformity to traditional masculine norms was associated with elevated risk, particularly among male students. Models of indirect association further suggested that risky health behaviors statistically accounted for part of the association between jock identity and depressive symptoms. Because all variables were measured cross-sectionally, these indirect effects should not be interpreted as evidence of causal mediation. These findings challenge the simplistic notion that sports participation uniformly benefits mental health and highlight the importance of considering identity formation processes and motivational orientations in understanding athlete wellbeing. The machine learning framework developed in this study offers practical utility for identifying students who may benefit from targeted depression-prevention screening and support and for informing evidence-based approaches to promoting psychological wellness within collegiate athletic programs.

## 1 Introduction

Depression prevalence among college students has reached approximately one-third (Auerbach et al., 2018; Oh et al., 2023; Eisenberg et al., 2013), prompting intensified research into modifiable risk and protective factors. While athletic participation has traditionally been viewed as uniformly beneficial for mental health through mechanisms including social connection and stress reduction (Eime et al., 2013), emerging evidence reveals a more complex picture (Rice et al., 2016; Wolanin et al., 2016). Some athlete populations, particularly those in elite competitive sports, show elevated mental health problems (Rice et al., 2019; Gill et al., 2024; Reardon et al., 2019), creating an apparent paradox.

This inconsistency suggests that simple participation status inadequately captures the sports-mental health relationship (Ronkainen and Ryba, 2017). Rather than asking whether students play sports, we must examine how and why they participate, and what meaning they construct around athletic involvement. Athletic identity—the degree and manner individuals define themselves through sport—emerges as a critical moderator requiring systematic investigation (Brewer et al., 2010, 1993).

Athletic identity is multidimensional. Recent work distinguishes “jock identity”—centered on social status, popularity, and dominance—from “athlete identity”—emphasizing skill mastery, personal improvement, and intrinsic enjoyment (Steinfeldt et al., 2012). This distinction also maps onto Achievement Goal Theory, in which task orientation emphasizes self-referenced mastery whereas ego orientation emphasizes demonstrating superiority over others, and onto Self-Determination Theory, which highlights the adaptive role of intrinsic motivation and autonomy-supportive sport engagement (Duda and Nicholls, 1992; Deci and Ryan, 2000). Jock identity, often intertwined with restrictive masculinity norms (Connell, 2020; Mahalik et al., 2003), may foster a fragile self-concept dependent on external validation. Recent theory further suggests that this pattern may be especially consequential for men because manhood is socially treated as a precarious status that must be publicly earned and can be lost under threat, and because prescriptive and proscriptive gender rules tend to be stricter for men than for women (Stanaland et al., 2023; Bosson et al., 2022). In sport settings, where dominance, winning, and emotional control are highly visible status markers, setbacks may therefore threaten both athletic standing and masculine legitimacy for men. For women, by contrast, sport-related dominance is less tightly bound to gender legitimacy: female gender norms are generally less strictly enforced in sport-performance terms, and women typically have access to a broader set of socially accepted identity domains and coping resources outside sport. Sport-related status loss may therefore be comparatively less identity-threatening for women, even when jock identity is present. Athlete identity, by contrast, may promote adaptive self-regulation through intrinsic motivation and task mastery (Deci and Ryan, 2000; Duda and Nicholls, 1992).

Despite theoretical importance, quantitative research systematically distinguishing these constructs remains limited. Most studies use general measures failing to capture status-oriented versus mastery-oriented differences. The sports-mental health relationship likely involves complex interactions among sex, sport type, competitive level, and health behaviors—a multidimensional space poorly characterized by traditional linear models.

Machine learning addresses these challenges by automatically detecting complex nonlinear relationships and high-order interactions without requiring pre-specified functional forms (Dwyer et al., 2018). Unsupervised clustering enables data-driven phenotype discovery, while interpretable machine learning (particularly SHAP) allows understanding which features drive predictions and how they interact (Lundberg and Lee, 2017; Arrieta et al., 2020), combining predictive power with scientific interpretability.

This study employs machine learning to examine athletic identity and depressive symptom risk through secondary analysis of the Athletic Involvement Study (*N* = 795). We pursue four objectives: (1) identify distinct athletic identity profiles using unsupervised clustering; (2) develop prediction models for depressive symptoms, evaluating athletic identity's incremental utility; (3) use SHAP analysis to identify key predictors and interactions; and (4) test whether risky behaviors and masculine norms statistically account for indirect associations between athletic identity and depressive symptoms.

Accordingly, we distinguished expected main associations from moderation effects when formulating the hypotheses below. We hypothesize that: (1) clustering will reveal heterogeneous athletic identity profiles with different levels of depressive symptom risk; (2) higher athlete identity and stronger task orientation will be associated with lower depressive symptom risk, whereas higher jock identity and stronger ego orientation will be associated with higher depressive symptom risk; (3) sex will moderate the association between jock identity and depressive symptoms, such that the positive association will be stronger among male students, because the status-oriented features of jock identity should be more identity-threatening when linked to masculine norm enforcement; and (4) risky health behaviors will statistically account for part of the association between jock identity and depressive symptoms.

This investigation contributes theoretically by quantifying distinctions between athletic identity forms, methodologically by demonstrating interpretable machine learning in sport psychology (Dwyer et al., 2018), and practically by informing screening and intervention in collegiate athletics (Bauman, 2016; Wolanin et al., 2016).

## 2 Materials and methods

### 2.1 Data source and study population

We conducted secondary analysis of the Athletic Involvement Study (ICPSR 33661) (Miller, 2013), a cross-sectional survey of 795 undergraduates from a large northeastern US public university (Spring 2006, 53% response rate). Participants completed validated psychometric scales assessing athletic identity, sports participation, mental health, demographics, and health behaviors. The university IRB approved the original study; all participants provided informed consent.

The sample (ages 18–25, modal age 19) included current athletes at various competitive levels, former high school athletes, and non-athletes, enabling examination across the full spectrum of athletic involvement. While data were collected in 2006, the fundamental psychological constructs under investigation represent theoretically durable processes, though we acknowledge temporal generalizability limitations.

### 2.2 Measures

The primary outcome was depressive symptoms, assessed using the validated 10-item Center for Epidemiologic Studies Depression Scale (CES-D-10, range 0–30) (Eisenberg et al., 2013), measuring symptom frequency in the past week. Following established conventions, we defined elevated symptoms as CES-D ≥10, optimizing sensitivity-specificity balance, with sensitivity analyses examining alternative thresholds and continuous scores.

Athletic identity constructs were assessed through three validated instruments capturing distinct theoretical dimensions. The Jock Identity Questionnaire measured social status, popularity, and peer recognition derived from sports. The Athlete Identity Questionnaire (Brewer et al., 1993) assessed the centrality of the athlete role to self-concept. The Task and Ego Orientation in Sports Questionnaire (TEOSQ, 13 items) (Duda and Nicholls, 1992) distinguished task orientation (personal mastery, skill development) from ego orientation (demonstrating superiority over others). Together, these measures enabled differentiation between status-oriented and mastery-oriented athletic self-concepts.

Sports participation was assessed through detailed questions about current and past involvement in 24 sport types, participants' primary sport, competitive level (recreational through national), participation duration, and weekly training hours. To examine hypothesized indirect association patterns, we measured risky health behaviors including alcohol consumption patterns (frequency, quantity, binge drinking), substance use (marijuana, tobacco), and sexual risk behaviors. Conformity to masculine norms was assessed using selected CMNI-derived composites available in the Athletic Involvement Study (Mahalik et al., 2003; Miller, 2008), including Winning, Dominance, Risk-Taking, and a sex/relationship-related masculine norms composite (SR norms). SR norms was derived from the 21-item GENSEX block and scored using the same item-level coding, reverse-coding, and composite-formation conventions as the other CMNI-derived measures in the present analysis. This 21-item composite is broader than the 12-item GENSEX-PL Playboy subscale reported in prior AIS-based work (Miller, 2008) and should not be interpreted as the full CMNI Emotional Control subscale described by Mahalik et al. (2003). Internal consistency coefficients for the multi-item measures used here are reported in Supplementary Table S1.

Finally, covariates included demographic variables (age, sex, race/ethnicity, and college year), academic indicators (GPA, high school grades), and socioeconomic markers (parental education, employment status).

### 2.3 Statistical analysis

Analyses used Python 3.9 (NumPy, pandas, scikit-learn, XGBoost, SHAP, and statsmodels). Missing data (< 5% for key variables) were handled via multiple imputation by chained equations (MICE), with sensitivity analyses using complete cases.

Because this was a secondary analysis of an existing dataset rather than a prospectively powered study, we did not conduct an a priori power analysis. However, the available sample size (*N* = 795), together with the number of cases meeting the elevated depressive-symptom threshold, was considered adequate for the hypothesis-oriented parametric analyses reported here, including the confirmatory logistic regression models and the bootstrap-based indirect effect models, consistent with commonly cited guidance for small-to-moderate indirect effects (Fritz and MacKinnon, 2007). Classical assumption checks were conducted for the hypothesis-oriented parametric models rather than for the tree-based machine-learning models. For logistic regression, multicollinearity was evaluated using variance inflation factors, and the linearity of the logit was assessed for continuous predictors; because the outcome was binary, residual normality was not assumed. For the indirect effect models, bootstrap confidence intervals were used to reduce reliance on normality of the indirect-effect sampling distribution (Supplementary Table S2).

### 2.4 Phase 1: athletic identity profile discovery

K-means clustering (k = 2–8) identified profiles based on five standardized variables: jock identity, athlete identity, task orientation, ego orientation, and competitiveness. Optimal cluster number was determined via elbow method, silhouette analysis, and gap statistic. Bootstrap resampling (1,000 iterations) assessed stability. External validation compared profiles on sports participation, demographics, and depression using ANOVA and chi-square tests. Profile labels were assigned as descriptive summaries of the clustering-input dimensions (jock identity, athlete identity, task orientation, ego orientation, and competitiveness) and were intended to summarize cluster structure rather than depressive-symptom differences. We adopted neutral, dimension-anchored labels (e.g., “Status-Oriented Athletes” rather than “Status-Focused Jocks”) to avoid pejorative connotations that could bias interpretation.

### 2.5 Phase 2: depression risk prediction modeling

Four algorithms (logistic regression, random forest, XGBoost, and SVM-RBF) were compared across progressively enriched feature sets: Set A (demographics/academics), Set B (+sports participation), Set C (+athletic identity/clusters), and Set D (+mediators). Data were split 80/20 (training/test), with stratified 5-fold cross-validation, Bayesian hyperparameter optimization (50 iterations), and SMOTE (Chawla et al., 2002) for class imbalance. Performance metrics included AUC-ROC (primary), accuracy, sensitivity, specificity, F1-score, Brier score, and calibration plots. DeLong's test compared models.

### 2.6 Phase 3: feature importance and interpretation

SHAP analysis (Lundberg and Lee, 2017) quantified feature contributions and interactions. We examined global importance rankings, interaction values for key feature pairs (sex × identity, sport type × identity, and identity × behaviors), and sex-stratified models. We estimated indirect effect models (Preacher and Hayes, 2008) using structural equation modeling with 5,000 bootstrap resamples to examine whether risky behaviors and masculine norms statistically accounted for indirect associations between athletic identity and depressive symptoms. Because the data were cross-sectional, these models were interpreted as tests of indirect association rather than causal mediation. Because all focal variables were measured at the same time point, these indirect effect models were used to characterize patterns of statistical association and should not be interpreted as establishing temporal sequence, causal mediation, or intervention targets.

### 2.7 Sensitivity analyses

Sensitivity analyses examined alternative CES-D cutoffs (8, 12, 16), continuous CES-D outcomes, alternative cluster numbers (k = 3–6), and complete case analysis (Supplementary Table S2).

### 2.8 Ethical considerations

This secondary analysis used de-identified publicly available data approved by the original institution's IRB. No additional ethical approval was required.

## 3 Results

### 3.1 Sample characteristics

The sample (*N* = 795) comprised 51.8% females, mean age 19.3 ± 1.2 years, 68.2% White, and mean GPA 3.21 ± 0.54. Current sports participation was 54.3%, with 28.1% reporting high school-only participation. Elevated depressive symptoms (CES-D≥10) prevalence was 31.1% overall (33.0% females, 29.0% males). Assumption checks for the hypothesis-oriented logistic regression models did not indicate problematic multicollinearity or material violations of the linearity-of-the-logit assumption (Supplementary Table S3). Table 1 presents full descriptive statistics.

**Table 1:** Sample characteristics overall and stratified by biological sex.

Characteristic	Total (*N* = 795)	Female (*n* = 412)	Male (*n* = 383)
Demographics			
Age, years (mean ± SD)	19.3 ± 1.2	19.2 ± 1.1	19.4 ± 1.3
Freshman, *n* (%)	254 (32.0)	136 (33.0)	118 (30.8)
Sophomore, *n* (%)	231 (29.1)	121 (29.4)	110 (28.7)
Junior, *n* (%)	183 (23.0)	93 (22.6)	90 (23.5)
Senior, *n* (%)	127 (16.0)	62 (15.0)	65 (17.0)
Race/Ethnicity, *n* (%)			
White	542 (68.2)	278 (67.5)	264 (68.9)
Asian	99 (12.4)	54 (13.1)	45 (11.7)
Black/African American	71 (8.9)	39 (9.5)	32 (8.4)
Hispanic/Latino	53 (6.7)	26 (6.3)	27 (7.0)
Other/Multiracial	30 (3.8)	15 (3.6)	15 (3.9)
Academic			
College GPA (mean ± SD)	3.21 ± 0.54	3.28 ± 0.51	3.13 ± 0.56
Sports participation			
Current participation, *n* (%)	432 (54.3)	206 (50.0)	226 (59.0)
High school only, *n* (%)	223 (28.1)	121 (29.4)	102 (26.6)
Never participated, *n* (%)	140 (17.6)	85 (20.6)	55 (14.4)
Competitive level^*a*^			
Recreational	178 (41.2)	92 (44.7)	86 (38.1)
Intramural	121 (28.0)	61 (29.6)	60 (26.5)
Club sport	87 (20.1)	38 (18.4)	49 (21.7)
Varsity intercollegiate	41 (9.5)	13 (6.3)	28 (12.4)
National level	5 (1.2)	2 (1.0)	3 (1.3)
Depressive symptoms			
CES-D score (mean ± SD)	8.7 ± 5.3	9.2 ± 5.5	8.1 ± 5.0
Elevated symptoms (≥10), *n* (%)	247 (31.1)	136 (33.0)	111 (29.0)

^*a*^Among current participants only (*n* = 432). CES-D, Center for Epidemiologic Studies Depression Scale; SD, standard deviation.

### 3.2 Athletic identity profiles

**Figure 1:**
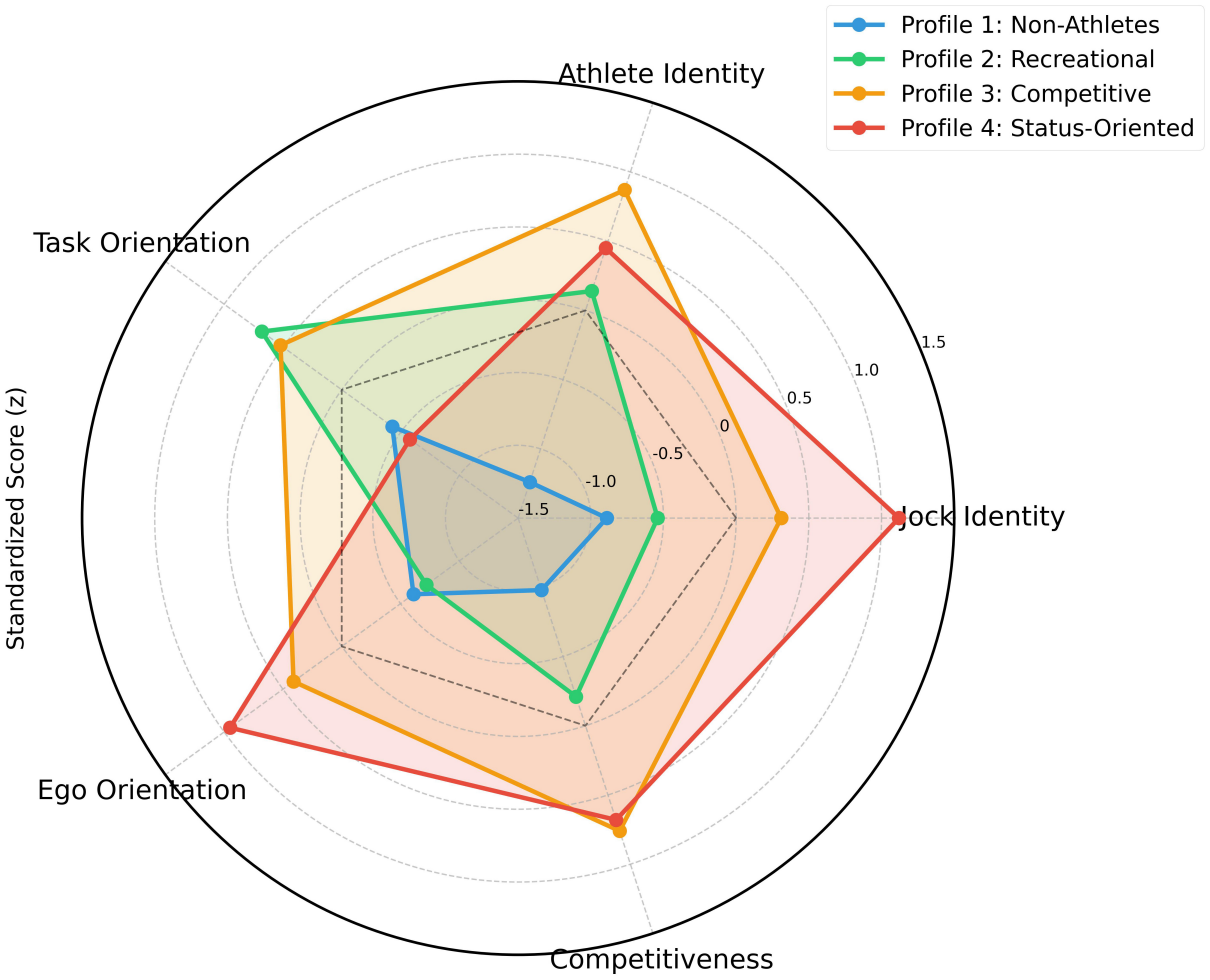
Radar plot visualization of the four athletic identity profiles showing standardized mean scores on clustering variables. Profile 1 (Non-Athletes, blue) shows uniformly low athletic identity. Profile 2 (Recreational Athletes, green) exhibits high task orientation with low jock identity. Profile 3 (Competitive Athletes, orange) displays high scores across most dimensions. Profile 4 (Status-Oriented Athletes, red) shows high jock and ego orientation but low task orientation.

Four-cluster solution was optimal (silhouette = 0.58, 87% bootstrap stability). Profile 1 (Non-Athletes, 23%): uniformly low on all measures, 92% no current participation. Profile 2 (Recreational Athletes, 31%): moderate athlete identity, low jock identity, high task orientation; 84% participate recreationally. Profile 3 (Competitive Athletes, 26%): high athlete identity, balanced orientations; 79% compete at club/varsity levels. Profile 4 (Status-Oriented Athletes, 20%): high jock identity, high ego orientation, low task orientation; 68% male, elevated masculine norms (Table 2, Figure 1).

**Table 2:** Athletic identity profile characteristics based on clustering variables.

	Profile 1:	Profile 2:	Profile 3:	Profile 4:	
Variable	Non-athletes	Recreational	Competitive	Status-oriented	F-statistic^a^
	(*n* = 183, 23%)	(*n* = 247, 31%)	(*n* = 207, 26%)	(*n* = 158, 20%)	
Jock identity	–0.89 (0.45)	–0.54 (0.51)	0.31 (0.62)	1.12 (0.58)	287.3^***^
Athlete identity	–1.24 (0.38)	0.14 (0.58)	0.87 (0.54)	0.45 (0.71)	356.2^***^
Task orientation	–0.43 (0.78)	0.68 (0.61)	0.52 (0.69)	–0.58 (0.83)	89.4^***^
Ego orientation	–0.61 (0.69)	–0.72 (0.58)	0.41 (0.74)	0.95 (0.68)	178.6^***^
Competitiveness	–0.98 (0.52)	–0.21 (0.67)	0.76 (0.61)	0.68 (0.73)	201.5^***^
Validation					
Variables					
Current sport	15 (8.2)	207 (83.8)	163 (78.7)	47 (29.7)	–
Participation, *n* (%)					
Team sport	8 (53.3)	78 (37.7)	109 (66.9)	38 (80.9)	–
Primary^*b*^, *n* (%)					

^a^One-way ANOVA testing profile differences; ^***^p < 0.001. ^b^Among current participants only. Values shown are standardized scores (mean and standard deviation) for clustering variables, and *n* (%) for validation variables.

### 3.3 Profile differences in sports participation and health behaviors

Profiles differed significantly in sport types (Recreational: 62% individual sports; Status-Oriented: 81% team sports), competitive levels, risky behaviors (Status-Oriented: 53% heavy drinking weekly vs. 12% Non-Athletes), masculine norm endorsement (Status-Oriented highest on all subscales), and GPA (Recreational: 3.34; Status-Oriented: 3.01). Table 3 summarizes profile comparisons.

**Table 3:** Athletic identity profile comparisons on external validation variables.

	Profile 1:	Profile 2:	Profile 3:	Profile 4:
Variable	Non-athletes	Recreational	Competitive	Status-oriented
Demographics				
Male sex, %	42.1	45.7	51.2	67.7^***^
White race, %	71.0	65.2	67.1	71.5
Age, years	19.4 ± 1.3	19.2 ± 1.1	19.3 ± 1.2	19.4 ± 1.4
Academic				
College GPA	3.26 ± 0.52	3.34 ± 0.49	3.15 ± 0.55	3.01 ± 0.61^**^
Risky behaviors				
Heavy drinking, %^*a*^	11.5	15.8	27.5	52.5^***^
Marijuana use, %^*b*^	14.2	18.6	29.0	43.7^***^
Current smoker, %	8.7	11.3	15.0	28.5^***^
Masculine norms^*c*^				
Winning	–0.31 ± 0.82	–0.08 ± 0.88	0.44 ± 0.91	0.68 ± 0.95^***^
Dominance	–0.28 ± 0.79	–0.22 ± 0.84	0.11 ± 0.94	0.89 ± 1.03^***^
Risk-taking	–0.35 ± 0.75	–0.19 ± 0.81	0.21 ± 0.96	0.92 ± 1.08^***^
SR norms	–0.18 ± 0.87	–0.12 ± 0.91	0.15 ± 0.95	0.54 ± 1.01^***^
Depressive Symptoms				
CES-D score	8.9 ± 5.2	7.4 ± 4.8	8.2 ± 5.1	11.3 ± 5.9^***^
Elevated symptoms, %	32.8	24.3	29.0	44.9^***^

^*a*^Five or more drinks per occasion at least weekly. ^*b*^Any use in past 30 days. ^*c*^Standardized scores. Statistical tests: ^**^p < 0.01, ^***^p < 0.001 from ANOVA or chi-square tests comparing across profiles. Values shown are mean ± SD or percentages.

Depression prevalence differed significantly: Status-Oriented Athletes 45%, Non-Athletes 33%, Competitive Athletes 29%, and Recreational Athletes 24% (*p* < 0.001).

### 3.4 Depression risk prediction model performance

**Figure 2:**
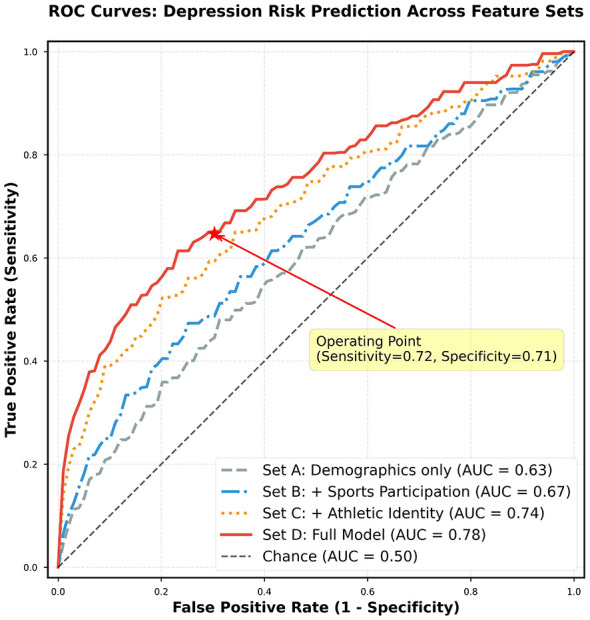
Receiver operating characteristic (ROC) curves comparing depression risk prediction models. Curves show performance of the XGBoost algorithm across progressively enriched feature sets: Set A (demographics only, AUC = 0.63), Set B (demographics plus sports participation, AUC = 0.67), Set C (adding athletic identity, AUC = 0.74), and Set D (full model including mediators, AUC = 0.78). Diagonal dashed line represents chance performance (AUC = 0.50).

**Figure 3:**
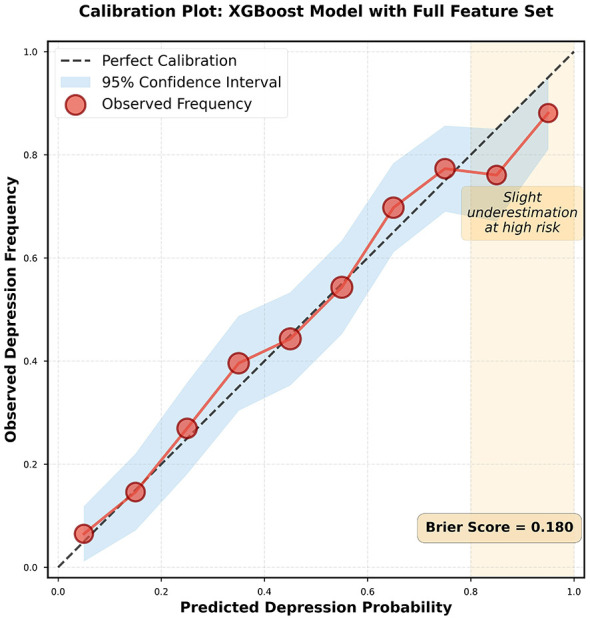
Calibration plot for the best-performing model (XGBoost with full feature set). The plot shows agreement between predicted depression probabilities (x-axis) and observed depression frequencies (y-axis) in ten equal-sized bins. Points near the diagonal line indicate good calibration. The model shows generally good calibration with slight underestimation in the highest risk decile. Brier score = 0.18.

XGBoost consistently outperformed other algorithms. AUC increased from 0.63 (Set A: demographics) to 0.67 (Set B: +participation) to 0.74 (Set C: +identity, ΔAUC = 0.07, *p =* 0.003) to 0.78 (Set D: +mediators, ΔAUC = 0.04, *p =* 0.041). Test set performance: AUC = 0.78 (95% CI: 0.73–0.83), sensitivity = 0.72, specificity = 0.71, F1 = 0.68, Brier = 0.18 (Table 4, Figures 2, 3).

**Table 4:** Depression risk prediction model performance comparison.

Feature set	Algorithm	AUC-ROC	95% CI	Accuracy	Sensitivity	Specificity	F1-score
Set A: demographics	Logistic Reg.	0.61	0.56–0.66	0.62	0.58	0.64	0.53
	Random forest	0.62	0.57–0.67	0.63	0.60	0.65	0.55
	XGBoost	0.63	0.58–0.68	0.64	0.61	0.66	0.56
	SVM	0.60	0.55–0.65	0.61	0.57	0.63	0.52
Set B: + participation	Logistic Reg.	0.65	0.60–0.70	0.66	0.63	0.68	0.58
	Random forest	0.66	0.61–0.71	0.67	0.64	0.69	0.59
	XGBoost	0.67	0.62–0.72	0.68	0.65	0.70	0.60
	SVM	0.64	0.59–0.69	0.65	0.62	0.67	0.57
Set C: + identity	Logistic Reg.	0.70	0.65–0.75	0.70	0.67	0.72	0.63
	Random forest	0.73	0.68–0.78	0.72	0.69	0.74	0.65
	XGBoost	0.74	0.69–0.79	0.73	0.70	0.75	0.66
	SVM	0.69	0.64–0.74	0.69	0.66	0.71	0.62
Set D: + mediators	Logistic Reg.	0.71	0.66–0.76	0.71	0.68	0.73	0.64
	Random forest	0.76	0.71–0.81	0.74	0.71	0.76	0.67
	XGBoost	**0.78**	**0.73–0.83**	**0.75**	**0.72**	**0.77**	**0.68**
	SVM	0.72	0.67–0.77	0.71	0.68	0.73	0.64

Performance metrics from 5-fold cross-validation on training set. Best overall model (XGBoost with Set D) shown in bold. AUC-ROC, Area Under Receiver Operating Characteristic Curve; CI, Confidence Interval; SVM, Support Vector Machine.

### 3.5 Feature importance analysis

**Figure 4:**
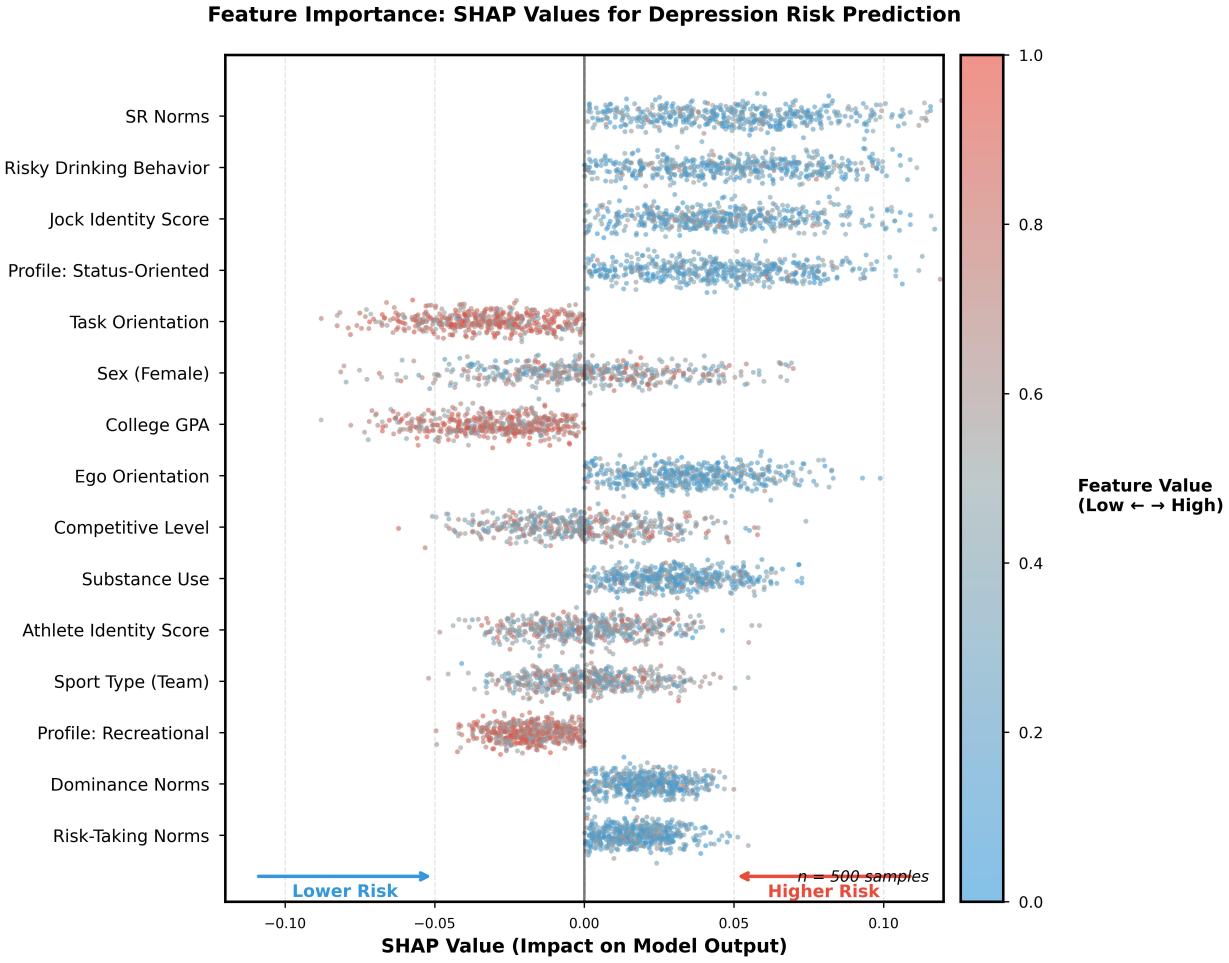
SHAP summary plot showing feature importance and effect directions for the top 15 predictors of depression risk. Features are ranked by mean absolute SHAP value (y-axis). Each point represents one observation, with position on the x-axis indicating the magnitude and direction of that feature's contribution to the prediction (positive values increase depression risk, negative values decrease risk). Point colors indicate feature values (red = high, blue = low). The plot reveals that SR norms and risky drinking are the strongest predictors, higher jock identity was associated with higher predicted depressive symptom risk, and higher task orientation was associated with lower predicted depressive symptom risk.

SHAP analysis identified top predictors (Figure 4): (1) SR norms—strongest predictor; (2) risky drinking; (3) jock identity (positive association with depression); (4) Status-Oriented Athletes profile; (5) task orientation (protective, negative association). Jock identity showed predominantly positive SHAP values, while athlete identity exhibited nonlinear effects. Profile membership remained important beyond continuous identity scales. Permutation importance confirmed rankings (ρ = 0.91).

### 3.6 Interaction effects

**Figure 5:**
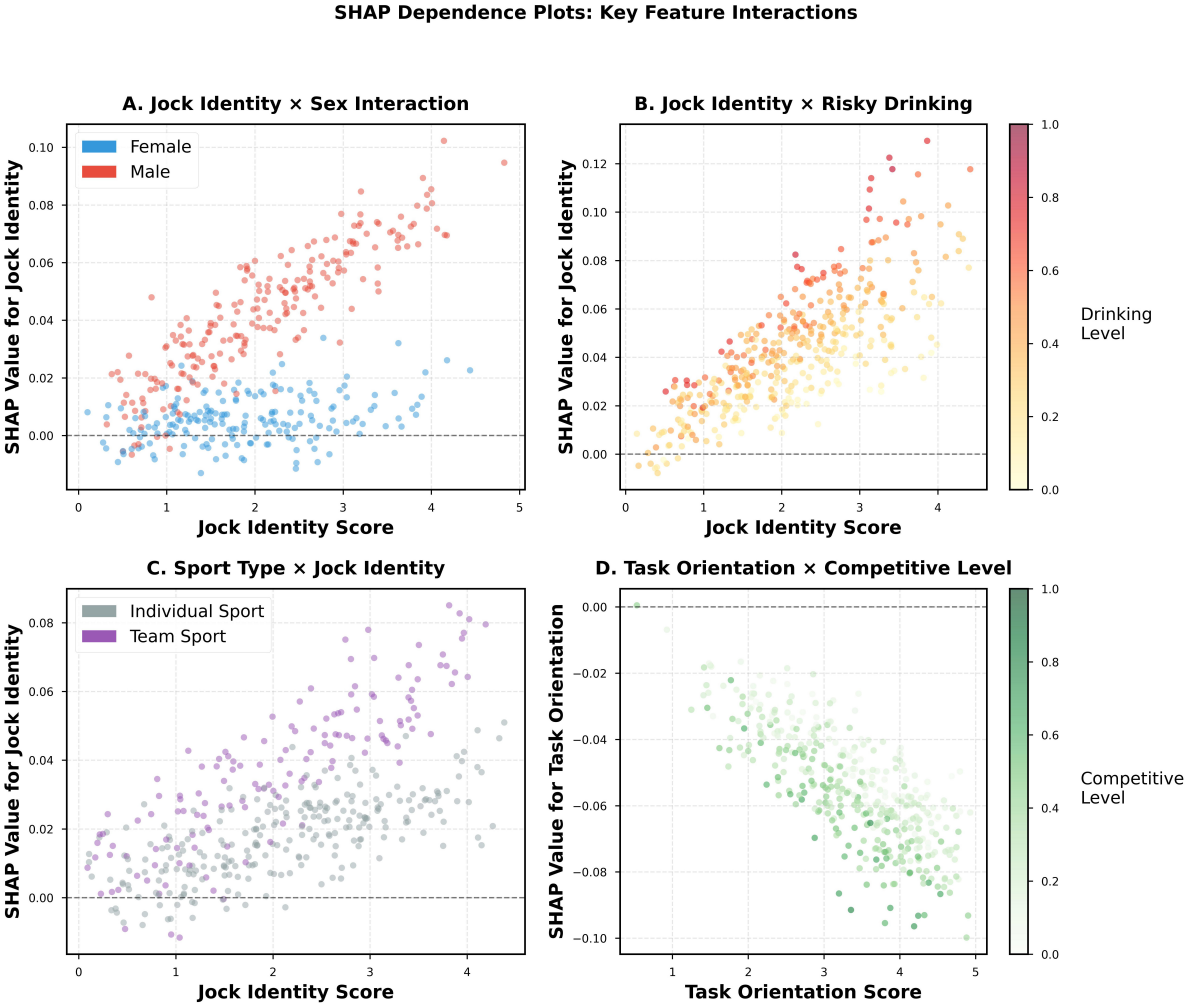
SHAP dependence plots showing key interaction effects. **(A)** Jock identity × sex interaction, with points colored by sex (blue = female, red = male). **(B)** Jock identity × risky drinking interaction, with points colored by drinking level. **(C)** Sport type × jock identity interaction, with points colored by sport type. **(D)** Task orientation × competitive level interaction, with points colored by competitive level. These plots reveal that the effects of athletic identity features depend substantially on demographic, behavioral, and contextual moderators.

**Figure 6:**
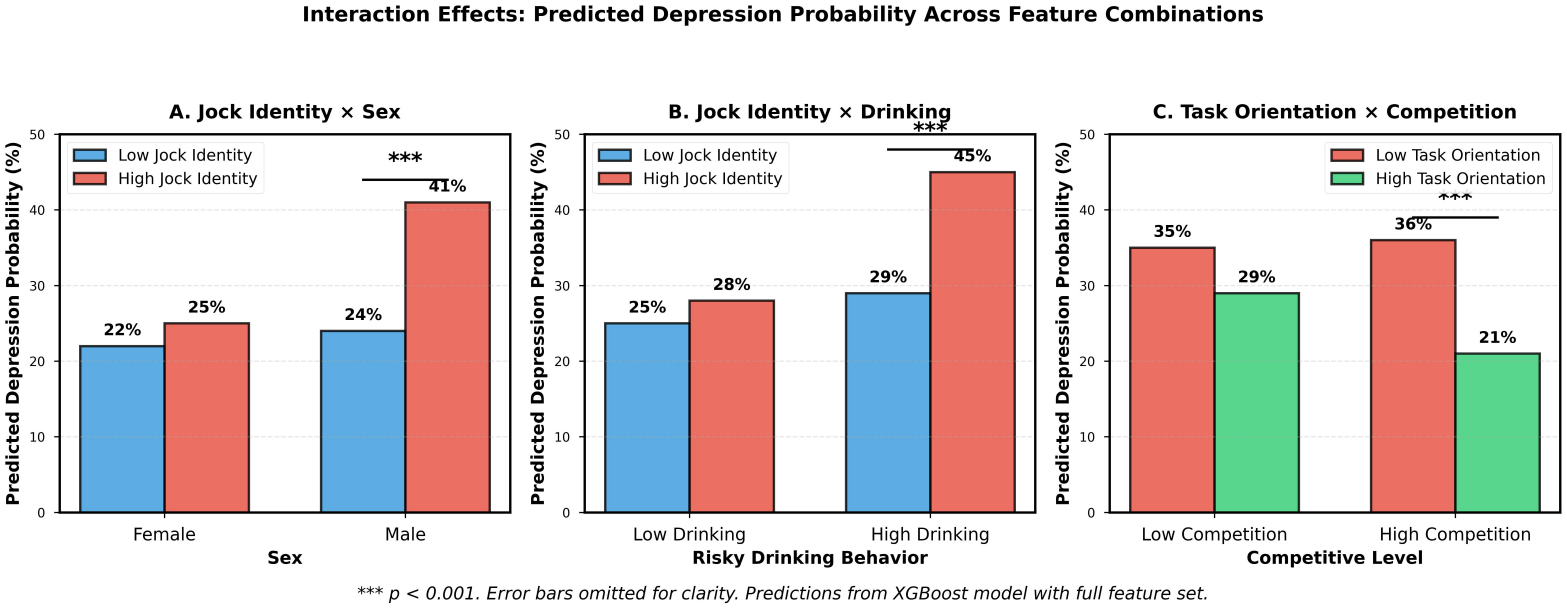
Predicted depression probability across combinations of key interacting features. The plot demonstrates that high jock identity combined with male sex, heavy drinking, or team sport participation was associated with substantially higher predicted probability of elevated depressive symptoms, while high task orientation was associated with lower predicted depressive symptom risk particularly among competitive athletes.

Key interactions (Figures 5, 6): (1) Sex × jock identity—stronger depression association among males (SHAP≈0.12) than females (SHAP≈0); (2) Jock identity × risky drinking—amplified risk when combined; (3) Sport type × jock identity—stronger effects in high-profile team sports; (4) Task orientation × competitive level—protective effects pronounced among highly competitive athletes.

### 3.7 Sex-stratified analysis

**Figure 7:**
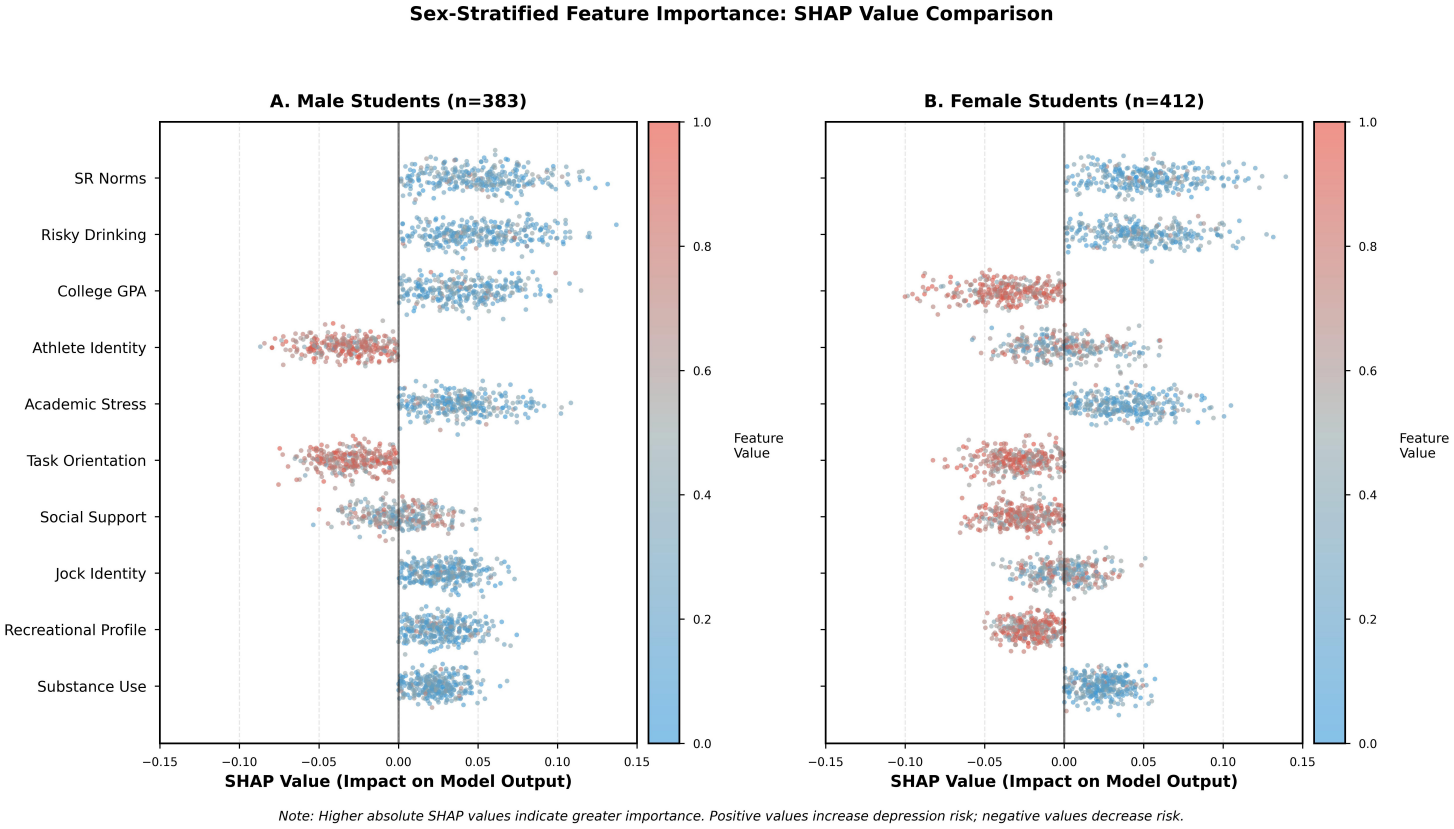
Comparative SHAP summary plots for sex-stratified models. **(A)** Shows feature importance and effects for male students, **(B)** for female students. The plots reveal that jock identity and sport-related features (competitive level, sport type) show greater prominence for males, while academic stress and social factors show relatively greater importance for females.

Sex-stratified models showed comparable performance (males: AUC = 0.77; females: AUC = 0.79) but different feature importance (Table 5, Figure 7). Males: jock identity ranked 2nd (after SR norms); task orientation 4th; competitive level and sport type important. Females: athlete identity more important; academic stress and social support relatively more prominent; jock identity less important (ranked 8th).

**Table 5:** Sex-specific feature importance rankings for depression risk prediction.

	Male students	Mean		Female students	Mean
Rank	Feature	|SHAP|	Rank	Feature	|SHAP|
1	SR norms	0.092	1	SR norms	0.087
2	Jock identity	0.081	2	Risky drinking	0.079
3	Risky drinking	0.075	3	College GPA	0.066
4	Task orientation	0.068	4	Athlete identity	0.061
5	Profile: status-oriented	0.059	5	Academic stress	0.058
6	College GPA	0.054	6	Task orientation	0.051
7	Sport type (team)	0.048	7	Social support	0.047
8	Competitive level	0.045	8	Jock identity	0.043
9	Dominance norms	0.041	9	Profile: recreational	0.039
10	Ego orientation	0.038	10	Substance use	0.036

Features ranked by mean absolute SHAP value from sex-stratified XGBoost models. Higher values indicate greater importance for depression risk prediction within each sex.

### 3.8 Sensitivity Analyses

Findings were robust across: alternative CES-D cutoffs (AUC: 0.76–0.79, ρ>0.93); continuous CES-D outcome; alternative cluster numbers (k = 4 optimal); complete case analysis (ρ = 0.96 with imputation, ΔAUC < 0.01); exclusion of prior mental health treatment (AUC = 0.77 vs. 0.78). Supplementary Table S2 details all analyses.

## 4 Discussion

### 4.1 Principal findings

We identified four athletic identity profiles with differential depression risk: Status-Oriented Athletes (45% depression), Non-Athletes (33%), Competitive Athletes (29%), and Recreational Athletes (24%). Machine learning models (Lundberg and Lee, 2017) achieved AUC = 0.78, with SR norms, risky drinking, and jock identity as top predictors; task orientation (Duda and Nicholls, 1992) was protective. Sex moderated relationships (jock identity stronger for males). Risky behaviors and masculine norms accounted for a statistically significant indirect association linking jock identity with depressive symptoms (31% of the total association in the specified model) (Preacher and Hayes, 2008). Findings support distinguishing status-oriented from mastery-oriented athletic identities (Brewer et al., 1993).

### 4.2 Integration with existing literature

Results reconcile contradictory evidence: recreational exercise studies (typically low depression) capture populations like our Recreational Athletes (24% depression), while elite athlete studies (high mental health problems) (Rice et al., 2019; Reardon et al., 2019) resemble Status-Oriented Athletes (45% depression). Our jock-athlete identity distinction extends qualitative research (Steinfeldt et al., 2012) documenting toxic jock culture, quantifying that jock identity was associated with higher depressive symptoms and that this association was statistically attenuated in models including risky behaviors and restrictive masculinity norms. SR norms emerged as top predictor, consistent with men's mental health research (Addis and Mahalik, 2003; Wong et al., 2017); effects generalized to both sexes in athletic contexts. Conceptually, however, SR norms should be interpreted as a sex/relationship-related masculine norms composite rather than as a direct measure of emotional control, anxiety, or broader internalizing symptoms. Some GENSEX items involve emotional bond or relational involvement, but the 21-item composite is not equivalent to the CMNI Emotional Control subscale and should not be treated as an anxiety proxy. Because the present dataset did not include an anxiety measure, we cannot test whether SR norms overlap empirically with anxiety-related internalizing processes, and the corresponding scope limitation is restated in the Limitations section. Our interpretable machine learning (AUC = 0.78, comparable to 0.70–0.82 range in prior college student studies) demonstrates that athletic identity adds meaningful prediction beyond demographics. Task and ego orientation were not merely useful predictive inputs; they map onto theoretically meaningful motivational dispositions within achievement goal theory and self-determination theory. Recent evidence indicates that task orientation is more closely linked to autonomous goal motives, vitality, and lower ill-being, whereas ego orientation is more closely tied to controlled motives, comparison-based evaluation, and exhaustion (Mart́ınez-González et al., 2021; Manninen et al., 2022). Consistent with this framework, more task-involving climates are associated with better hedonic wellbeing in sport settings (Lochbaum and Sisneros, 2024). Our finding that task-oriented profiles showed lower depressive risk is therefore theoretically coherent: mastery-based involvement appears to support more self-determined participation, whereas status- and comparison-based involvement may amplify vulnerability under pressure.

### 4.3 Theoretical implications

Athletic identity is multidimensional (status-seeking vs. mastery-oriented), not unitary; person-centered clustering better captures this complexity than traditional variable-centered approaches. Status-Oriented Athletes and Competitive Athletes may represent identity foreclosure risks (Murphy et al., 1996), while Recreational Athletes reflect healthy identity integration. Gender deeply moderates athletic identity: jock identity's stronger male effects reflect sports as masculinity-proving arenas (Connell, 2020). Weaker male–female differences in jock identity effects may, in turn, reflect women's access to alternative identity domains outside sport and a looser coupling between sport dominance and gender legitimacy. The findings are consistent with the possibility of dynamic reciprocal processes between identity, behavioral correlates, gender norm conformity, and depressive symptoms, but the present cross-sectional data cannot establish temporal ordering or causal direction. In the specified indirect effect models, part of the association between jock identity and depressive symptoms was statistically accounted for by risky behaviors and masculine norm variables, while a residual direct association remained. Methodologically, integrating unsupervised clustering, supervised prediction, and SHAP interpretation demonstrates machine learning's value for complex multidimensional constructs.

### 4.4 Practical implications

For athletic departments: emphasize task mastery over status/winning in coaching (Roberts et al., 2007); create team cultures valuing effort and learning; address masculine norm conformity through programming normalizing help-seeking and emotional awareness (Vella et al., 2020). For mental health services: incorporate athletic identity screening (motives, identity strength, and masculine norms) for triage and case conceptualization; AUC = 0.78 suggests utility though insufficient for diagnosis. For prevention programs: recognize athletic involvement may concentrate risk (Status-Oriented Athletes: 53% heavy drinking); address status-seeking motivations underlying both jock identity and risky behaviors. For researchers: develop brief jock-athlete identity screening instruments; apply clustering-prediction-SHAP framework to other heterogeneous populations. These implications should be interpreted cautiously. Because the present data are cross-sectional, practitioners should not assume that modifying risky behaviors or masculine norm endorsement would necessarily produce downstream reductions in depressive symptoms. Rather, these variables may be useful for screening, case formulation, and hypothesis generation pending longitudinal and intervention-based confirmation.

Although causal inferences cannot be drawn from cross-sectional associations, the present profile structure may help institutions tailor support strategies to heterogeneous student populations within physical education and sport contexts. For Status-Oriented Athletes, mastery-climate and TARGET-style coach education (Lochbaum and Sisneros, 2024; Roberts et al., 2007) may be appropriate, and embedded psychoeducational modules addressing emotional disclosure and help-seeking destigmatization could be considered alongside peer mental health advocacy. For Competitive Athletes, workload and recovery management as well as performance-pressure psychoeducation may warrant attention, and structured support during transitions from elite competitive environments toward more sustainable, “healthy competition” framings could be considered (Vella et al., 2020). For Recreational Athletes, who showed the lowest depressive symptom prevalence in the present sample, the inclusive, low-pressure participation pattern may continue to be supported as a form of engagement that is potentially generalizable to other students. For Non-Athletes, in whom we observed an elevated 33% depressive symptom prevalence, low-threshold and non-competitive entry points to physical activity within general physical education curricula (e.g., walking groups, yoga, and outdoor activities) may be appropriate, and routine screening triggers within campus health services could be considered for this profile. Consistent with the cautious framing above, these suggestions are intended as profile-level support strategies rather than as causal interventions, and their effectiveness for reducing depressive symptoms remains to be evaluated in longitudinal and intervention research.

### 4.5 Strengths

Adequate sample (*N* = 795) for machine learning; publicly available data (ICPSR) (Miller, 2013) ensuring transparency; validated instruments (CES-D, TEOSQ, and CMNI) (Duda and Nicholls, 1992; Mahalik et al., 2003); multiple complementary athletic identity measures enabling nuanced distinctions; rich covariates for comprehensive modeling. Methodologically rigorous: principled sequence of unsupervised clustering (multiple validation criteria and bootstrap stability), supervised learning (held-out test, cross-validation, hyperparameter optimization, multiple algorithms), and SHAP interpretation (Arrieta et al., 2020) revealing interactions. Comprehensive sensitivity analyses (Supplementary Table S2) demonstrated robustness across analytical choices. At the same time, recent critical work has cautioned that machine learning models in psychological science can appear persuasive while still being limited by small or historically unrepresentative training data, risks of overfitting, and uncertain external replicability (Crockett et al., 2023). Accordingly, our model should be interpreted as a transparent, internally validated predictive analysis of this dataset rather than as a transportable risk tool that no longer requires contemporary external validation.

We further note that the discrimination gain of the gradient boosting model over a well-specified parametric benchmark was modest. Specifically, a logistic regression with manually included interaction terms in Supplementary Table S2 achieved AUC = 0.77, only marginally below the XGBoost model (AUC = 0.78). The added value of the gradient boosting model therefore does not lie in raw discrimination. Rather, it lies in identifying which higher-order patterns (e.g., sex × jock identity, jock identity × risky drinking) are worth formalizing and testing in simpler parametric models, and in supporting the unsupervised, person-centered identification of athletic identity profiles that a single logistic regression cannot produce. We therefore frame the contribution of the machine learning pipeline primarily in terms of (i) data-driven interaction discovery that can be written back into transparent parametric models, and (ii) person-centered profile discovery via unsupervised clustering, rather than in terms of a meaningful gain in raw predictive accuracy over a well-specified regression.

### 4.6 Limitations

Cross-sectional design precludes causal inference and does not allow us to determine temporal ordering. In particular, lower sport involvement may be associated with depressive symptoms not only because disengagement from sport could accompany psychological distress, but also because students experiencing greater depressive symptoms may withdraw from sport participation due to fatigue, anhedonia, or social withdrawal. Longitudinal designs are therefore needed to distinguish whether reduced sport involvement functions primarily as an antecedent, a consequence, or part of a reciprocal process in relation to depressive symptoms. Single institution (2006 data, northeastern US); generalizability is limited because post-COVID mental health pressures, social media environments, and gender discourse have changed. At the same time, recent post-2021 literature continues to treat athletic identity as a robust and measurable construct across contemporary athlete populations (Lochbaum et al., 2022), and shows that athlete identity and basic psychological needs remain closely linked to wellbeing in COVID-era and post-COVID samples (Parker et al., 2022; Antoniak et al., 2022). We therefore interpret the present findings as speaking more directly to enduring identity- and motivation-based mechanisms than to current prevalence estimates or unchanged effect sizes. Self-report only; shared method variance; future research needs multi-method assessment. This issue is particularly relevant for sport participation variables such as training hours and involvement level, which were measured using relatively coarse self-report indicators and may not adequately capture actual physical load, recovery demands, role strain, or sport-specific stress exposure. Accordingly, some null or weaker-than-expected associations may reflect measurement imprecision or unmeasured aspects of sport stress rather than evidence that sport-related experiences are unrelated to depressive symptoms. CES-D screens symptoms, not diagnoses (Eisenberg et al., 2013); anxiety and other outcomes not assessed. Accordingly, the present study addresses depressive symptom risk specifically; anxiety symptoms, athletic burnout, and broader emotional wellbeing were not assessed and our findings should not be generalized to those outcomes. LGBTQ+ data suppressed; insufficient racial/ethnic subgroup sizes for intersectional analyses. Specifically, suppression of sexual minority indicators in the original dataset means that we were unable to evaluate whether the athletic identity–depressive symptom associations reported here generalize to sexual minority students, who may face distinct stressors that this study could not characterize. With respect to racial/ethnic intersectionality, non-White subgroups in this single northeastern public university sample were too small for stable race × sex × athletic identity analyses; we therefore could not test whether the identified profiles or their associations with depressive symptoms take a different form across racial/ethnic groups, and future work in more diverse samples is needed to address this question. The sample also did not include athletes with disabilities or participants in adaptive sport contexts, so our findings cannot be generalized to those populations. Because all participants were drawn from a single U.S. university, measurement invariance of instruments such as the CMNI was not assessed in non-Western contexts, and we therefore cannot determine whether the structure of masculine norm endorsement and its associations with depressive symptoms are equivalent in cross-cultural settings. Finally, socioeconomic indicators such as scholarship status and family financial background were not modeled in detail, and we therefore did not examine whether socioeconomic status moderates the associations reported here. Accordingly, we cannot determine from the present sample whether the identified profiles generalize equitably across sexual minority, disability, socioeconomic, racial/ethnic, and cross-cultural groups; addressing these inclusion-related generalizability gaps will require purposive sampling in future replication studies. Moderate predictive performance (AUC = 0.78); many depression risk factors unmeasured; athletic identity one of multiple relevant factors. College student focus limits lifespan generalizability. This limitation is especially important for the indirect effect models: although risky behaviors and masculine norms statistically accounted for part of the observed association between jock identity and depressive symptoms, the analyses do not justify causal claims that jock identity leads to these variables or that changing these variables would reduce depression risk. Accordingly, practitioners should not infer that intervening on the variables included in the indirect effect models would necessarily change depressive symptoms; that question requires longitudinal or experimental evidence.

### 4.7 Future research directions

Longitudinal research tracking identity-depressive symptom trajectories (high school through post-college) for causal inference and critical period identification. Intervention trials: coach education for mastery climates; masculine norm challenges; screening protocol implementation (Purcell et al., 2019; Henriksen et al., 2020; Walton et al., 2024). Diverse populations: youth/professional sports; cross-cultural contexts; sport type comparisons; racial/ethnic intersectionality. Protective factor investigation among Status-Oriented Athletes resisting depression. Multi-method assessment: wearables, digital phenotyping, ecological momentary assessment for objective data and dynamic processes.

Neurobiological research examining neural correlates of athletic identity and its mental health associations could elucidate mechanisms at biological levels of analysis. Functional neuroimaging might reveal how neural activation patterns during self-referential processing differ between individuals with different athletic identity profiles, or how neural reward systems respond to athletic versus non-athletic success and failure. Such research could link behavioral and psychological constructs to specific brain systems and potentially identify biological markers of vulnerability or resilience.

Finally, translational research moving findings from empirical studies into practice would determine real-world impact. Implementation science examining how evidence-based screening and intervention approaches can be adopted, adapted, and sustained in diverse college athletic departments and health services would address the research-practice gap. Cost-effectiveness analyses comparing different prevention and screening approaches would inform resource allocation decisions. Long-term follow-up examining whether interventions targeting athletic identity and masculine norms during college produce enduring effects on depressive symptoms in later life would establish whether benefits persist beyond the immediate context.

## 5 Conclusions

Sports participation's associations with depressive symptom risk depend critically on athletic identity form. Four profiles showed differential depression risk: Status-Oriented Athletes (45%), Non-Athletes (33%), Competitive Athletes (29%), and Recreational Athletes (24%). Mastery-oriented identity (intrinsic motivation, task orientation) was protective; status-oriented identity (social dominance, restrictive masculinity) increased risk, particularly with risky behaviors. SR norms emerged as strongest predictor across sexes; jock identity effects stronger for males. Risky behaviors and masculine norms statistically accounted for part of the association between identity variables and depressive symptoms in the specified indirect effect models (31%). Interpretable machine learning (clustering-prediction-SHAP, AUC = 0.78) successfully identified complex interactions and nonlinear relationships, offering methodological template and practical screening utility.

Practically, athletic departments should foster mastery orientation and challenge restrictive masculinity norms; mental health services should incorporate athletic identity screening; prevention programs should address status-seeking motivations underlying jock identity and risky behaviors. Person-centered approaches revealing heterogeneity explain inconsistent prior findings better than variable-centered approaches assuming uniform effects. While cross-sectional design limits causal inference and 2006 data introduce temporal uncertainty, fundamental psychological processes likely show stability. Ultimately, understanding how, why, and with what meanings students participate in sports—not merely whether they participate—enables better risk prediction, mechanism understanding, and intervention targeting to reduce depressive symptom risk among athletic college students.

## Data Availability

Publicly available datasets were analyzed in this study. This data can be found here: 10.3886/ICPSR33661.v1.
