# Supplementary material for: Athletic identity and depressive symptom risk in college students: a machine learning approach to identify at-risk profiles and protective correlates
Source: Front Psychol. 2026 Jun 5;17:1764766. doi: 10.3389/fpsyg.2026.1764766 (PMC13278907; doi:10.3389/fpsyg.2026.1764766)
Supplement: Supplementary file 1 [file Table_1.pdf]

Supplementary Materials

Supplementary Table S1. Internal Consistency of Multi-Item Measures in the Current Sample

Table S1: Internal consistency of multi-item measures in the current sample.

Measure	Items	Cronbach's α	Notes
CES-D-10	10	0.84	Used in present analyses
TEOSQ Task	7	0.81	Used in present analyses
TEOSQ Ego	6	0.79	Used in present analyses
Athlete Identity Questionnaire (AIQ)	10	0.83	Used in present analyses
Jock Identity Questionnaire (JAQ)	8	0.78	Used in present analyses
CMNI-derived Winning	6	0.76	Used in present analyses
CMNI-derived Dominance	6	0.74	Used in present analyses
CMNI-derived Risk-Taking	5	0.72	Used in present analyses
CMNI-derived SR norms (21-item GENSEX)	21	0.85	Used in present analyses[a]
CMNI Playboy subscale (12-item GENSEX-PL)	12	0.82	Comparability only[b]
CMNI Romantic relationship (9-item GENSEX-RR)	9	0.79	Transparency only[c]

[a]The 21-item SR norms composite (GENSEX01–GENSEX21) was the measure actually entered into the machine learning models and indirect effect analyses. This composite is broader than the 12-item Playboy subscale reported by Miller (2008) and is not equivalent to the full CMNI Emotional Control subscale described by Mahalik et al. (2003).

[b]The 12-item GENSEX-PL Playboy subscale is reported here for comparability with prior AIS-based work (Miller, 2008).

[c]The 9-item GENSEX-RR Romantic relationship subscale is reported for transparency regarding the composition of the 21-item SR norms composite.

Supplementary Table S2. Sensitivity Analysis Results

Alternative Depression Cutoffs We examined CES-D cutoff values ranging from 8 (more sensitive, identifying milder symptomatology) to 16 (more specific, focusing on moderate-to-severe symptoms). Model performance remained stable across cutoffs (AUC 0.76-0.79), and feature importance rankings showed high correlation ($\rho = 0.93$-0.96), indicating that findings are not artifacts of the specific threshold chosen. Analysis of continuous CES-D scores using gradient boosting regression (rather than classification) yielded similar patterns, with jock identity, SR norms, and risky drinking among the strongest predictors of higher depression scores.

Alternative Clustering Solutions The three-cluster solution collapsed the Competitive Athletes and Status-Oriented Athletes profiles into a single high athletic identity group, losing theoretically important distinctions and resulting in lower silhouette coefficient (0.52 vs. 0.58 for four clusters). Five- and six-cluster solutions produced additional splits that showed poor stability in bootstrap resampling (consistency $< 80\%$) and lacked clear theoretical interpretation. Depression prevalence differences between the additional sub-clusters in five- and six-cluster solutions were not statistically significant. The four-cluster solution optimally balanced statistical criteria, stability, and interpretability.

Table S2: Sensitivity Analyses: Model Performance and Key Findings Across Alternative Specifications

Analysis	Specification	N	AUC-ROC	95% CI	Key Finding	Consistency[a]
**Alternative Depression Cutoffs**						
Primary	CES-D ≥ 10	795	0.78	0.73-0.83	Status-Oriented Athletes highest risk	–
Alternative 1	CES-D ≥ 8	795	0.76	0.71-0.81	Status-Oriented Athletes highest risk	$\rho = 0.94$
Alternative 2	CES-D ≥ 12	795	0.79	0.74-0.84	Status-Oriented Athletes highest risk	$\rho = 0.96$
Alternative 3	CES-D ≥ 16	795	0.77	0.71-0.83	Status-Oriented Athletes highest risk	$\rho = 0.93$
Continuous	CES-D score (0-30)	795	$R^2 = 0.41$	0.36-0.46	Higher jock identity was associated with higher CES-D scores	$\rho = 0.95$
**Alternative Clustering Solutions**						
Primary	k = 4 clusters	795	0.78	0.73-0.83	Four profiles identified	–
Alternative 1	k = 3 clusters	795	0.74	0.69-0.79	Competitive/Status merged	Silhouette = 0.52
Alternative 2	k = 5 clusters	795	0.77	0.72-0.82	Profile 3 split, unstable	Silhouette = 0.54
Alternative 3	k = 6 clusters	795	0.76	0.71-0.81	Further splits, unstable	Silhouette = 0.49
**Missing Data Approaches**						
Primary	Multiple imputation (MICE)	795	0.78	0.73-0.83	All features included	–
Alternative	Complete case analysis	712	0.77	0.72-0.82	Consistent patterns	$\rho = 0.96$
Alternative	Listwise deletion	749	0.78	0.73-0.83	Minimal difference	$\rho = 0.98$
**Sample Restrictions**						
Primary	Full sample	795	0.78	0.73-0.83	All participants included	–
Alternative 1	Exclude prior MH treatment	708	0.77	0.72-0.82	Similar risk factors	$\rho = 0.97$
Alternative 2	Current participants only	432	0.76	0.69-0.83	Jock identity stronger effect	$\rho = 0.89$
Alternative 3	Never participated excluded	655	0.79	0.74-0.84	Among athletes only	$\rho = 0.92$
**Algorithm Comparisons (Full Feature Set D)**						
Primary	XGBoost	795	0.78	0.73-0.83	Best performance	–
Alternative 1	Random Forest	795	0.76	0.71-0.81	Comparable performance	$\rho = 0.94$
Alternative 2	Logistic Regression	795	0.71	0.66-0.76	Lower but adequate	$\rho = 0.88$
Alternative 3	SVM (RBF kernel)	795	0.72	0.67-0.77	Moderate performance	$\rho = 0.86$
Alternative 4	Neural Network (2 layers)	795	0.77	0.72-0.82	Good performance	$\rho = 0.91$
**Feature Importance Methods**						
Primary	SHAP values	795	–	–	Top 3: SRnorms, Drinking, Jock	–
Alternative 1	Permutation importance	795	–	–	Top 3: SRnorms, Jock, Drinking	$\rho = 0.91$
Alternative 2	MDI (Gini importance)	795	–	–	Top 3: Drinking, SRnorms, Jock	$\rho = 0.87$
**Interaction Specifications**						
Primary	SHAP interaction values	795	–	–	Sex × Jock strongest	–
Alternative 1	Manual interaction terms	795	0.77	0.72-0.82	Sex × Jock significant ($p<0.01$)	Consistent
Alternative 2	Stratified models by sex	795	–	–	Jock more important for males	Consistent
**Cross-Validation Schemes**						
Primary	5-fold stratified CV	795	0.78	0.73-0.83	Standard approach	–
Alternative 1	10-fold stratified CV	795	0.78	0.73-0.83	Minimal difference	ΔAUC < 0.01
Alternative 2	Leave-one-out CV	795	0.77	0.72-0.82	Computationally intensive	ΔAUC = 0.01
Alternative 3	Repeated 5-fold (3 reps)	795	0.78	0.74-0.82	More stable estimates	SD = 0.02
**Class Imbalance Approaches**						
Primary	SMOTE oversampling	795	0.78	0.73-0.83	Balanced training data	–
Alternative 1	No resampling	795	0.74	0.69-0.79	Lower sensitivity	Sens = 0.64
Alternative 2	Random oversampling	795	0.77	0.72-0.82	Similar to SMOTE	ΔAUC = 0.01
Alternative 3	Class weights only	795	0.76	0.71-0.81	Adequate performance	ΔAUC = 0.02
**Indirect Effect Model Specifications**						
Primary	Bootstrap 5000 resamples	795	–	–	31% indirect association accounted for by behavioral variables	–
Alternative 1	Bootstrap 10000 resamples	795	–	–	31% indirect association estimated, narrower CI	CI width ↓ 8%
Alternative 2	Normal theory SE	795	–	–	29% indirect association estimated, wider CI	CI symmetric
Alternative 3	Bias-corrected bootstrap	795	–	–	32% indirect association estimated	Consistent

[a]Consistency assessed via Spearman correlation (ρ) of feature importance rankings with primary analysis, or qualitative assessment. AUC-ROC = Area Under Receiver Operating Characteristic Curve; CI = Confidence Interval; CV = Cross-Validation; MICE = Multiple Imputation by Chained Equations; MH = Mental Health; SMOTE = Synthetic Minority Oversampling Technique; SVM = Support Vector Machine; SE = Standard Error; SRnorms = SR norms; MDI = Mean Decrease in Impurity.

Key Findings Summary: Across all sensitivity analyses, the core findings remained robust: (1) Status-Oriented Athletes profile consistently showed highest depression risk; (2) Jock identity, SR norms, and risky drinking consistently ranked among top predictors; (3) Task orientation showed protective effects across specifications; (4) Sex × Jock identity interaction remained significant; (5) Model performance (AUC 0.76-0.79) was stable across analytical choices. The four-cluster solution showed optimal balance of statistical quality, stability, and interpretability compared to alternative numbers of clusters. XGBoost showed best performance but Random Forest achieved comparable results. Multiple imputation and complete case analysis yielded nearly identical results, validating the imputation approach. Feature importance rankings correlated at $\rho > 0.85$ across different importance calculation methods, indicating robust identification of key predictors.

Supplementary Table S3. Assumption Checks for the Hypothesis-Oriented
Logistic Regression Models

Table S3: Assumption checks for the hypothesis-oriented logistic regression models.

**Predictor**	**Type**	**VIF**	**Linearity of Logit**	**Comment**
Age	Continuous	1.12	Satisfied	No concern
College GPA	Continuous	1.24	Satisfied	No concern
Jock Identity	Continuous	1.89	Satisfied	No concern
Athlete Identity	Continuous	1.94	Satisfied	No concern
Task Orientation	Continuous	1.67	Satisfied	No concern
Ego Orientation	Continuous	1.58	Satisfied	No concern
SR norms	Continuous	1.73	Satisfied	No concern
Winning	Continuous	1.61	Satisfied	No concern
Dominance	Continuous	1.82	Satisfied	No concern
Risk-Taking	Continuous	1.55	Satisfied	No concern
Risky Drinking	Continuous	1.48	Satisfied	No concern
Sex	Categorical	1.31	N/A	No concern
Race/Ethnicity	Categorical	1.18	N/A	No concern
Sport Type	Categorical	1.52	N/A	No concern
Competitive Level	Ordinal	1.44	Satisfied	No concern
Profile Membership	Categorical	2.21	N/A	Acceptable[a]

VIF = Variance Inflation Factor. Linearity of the logit was assessed using Box-Tidwell tests for continuous predictors; all continuous predictors satisfied the assumption (all $p > 0.05$). VIF values below 5 are generally considered acceptable; all predictors met this criterion, indicating no problematic multicollinearity.

[a]The slightly elevated VIF for profile membership reflects expected overlap with continuous identity variables used in clustering; this was not considered problematic because profile membership and continuous identity scales were not simultaneously included in the same model specifications.
